# An edge-directed interpolation method for fetal spine MR images

**DOI:** 10.1186/1475-925X-12-102

**Published:** 2013-10-10

**Authors:** Shaode Yu, Rui Zhang, Shibin Wu, Jiani Hu, Yaoqin Xie

**Affiliations:** 1 Shenzhen Institutes of Advanced Technology, Chinese Academy of Sciences, Shenzhen, China; 2 Shenzhen Key Laboratory for Low-cost Healthcare, Shenzhen, China; 3 Wayne State University, Detroit, MI, USA

**Keywords:** Magnetic resonance imaging, Fetal spine, Edge-directed interpolation

## Abstract

**Background:**

Fetal spinal magnetic resonance imaging (MRI) is a prenatal routine for proper assessment of fetus development, especially when suspected spinal malformations occur while ultrasound fails to provide details. Limited by hardware, fetal spine MR images suffer from its low resolution.

High-resolution MR images can directly enhance readability and improve diagnosis accuracy. Image interpolation for higher resolution is required in clinical situations, while many methods fail to preserve edge structures. Edge carries heavy structural messages of objects in visual scenes for doctors to detect suspicions, classify malformations and make correct diagnosis. Effective interpolation with well-preserved edge structures is still challenging.

**Method:**

In this paper, we propose an edge-directed interpolation (EDI) method and apply it on a group of fetal spine MR images to evaluate its feasibility and performance. This method takes edge messages from Canny edge detector to guide further pixel modification. First, low-resolution (LR) images of fetal spine are interpolated into high-resolution (HR) images with targeted factor by bi-linear method. Then edge information from LR and HR images is put into a twofold strategy to sharpen or soften edge structures. Finally a HR image with well-preserved edge structures is generated. The HR images obtained from proposed method are validated and compared with that from other four EDI methods. Performances are evaluated from six metrics, and subjective analysis of visual quality is based on regions of interest (ROI).

**Results:**

All these five EDI methods are able to generate HR images with enriched details. From quantitative analysis of six metrics, the proposed method outperforms the other four from signal-to-noise ratio (SNR), peak signal-to-noise ratio (PSNR), structure similarity index (SSIM), feature similarity index (FSIM) and mutual information (MI) with seconds-level time consumptions (TC). Visual analysis of ROI shows that the proposed method maintains better consistency in edge structures with the original images.

**Conclusions:**

The proposed method classifies edge orientations into four categories and well preserves structures. It generates convincing HR images with fine details and is suitable in real-time situations. Iterative curvature-based interpolation (ICBI) method may result in crisper edges, while the other three methods are sensitive to noise and artifacts.

## Background

Vertebral ossifications and most spinal defects are apparent about 20-22 weeks menstrual age. Ultrasound remains the primary technique for fetal spine imaging for its proven utility, widespread availability and relative low cost [1-3]. But disadvantages, like a small field of view, limited soft-tissue contrast and poor image quality in oligohydramnios, accordingly result in that ultrasound findings are occasionally inconclusive and insufficient to guide treatment choices [2-5].

Fetal MRI is an essential routine for prenatal examination, pregnancy care planning and postnatal facilitation. MR images are the first-hand materials for accurate diagnosis when fetus is with spine abnormalities, such as spinal dysraphism, spinal neoplasia and fetal myelomeningocoele. Fetal MRI provides efficient information for assessment, especially when suspected malformations occur while ultrasound fails to provide diagnosis details [2-8]. There are many factors accounting for the low visual quality of fetal spine MR images, including the inaccuracy determination of fetal position in uterus, the physicians’ limited understanding of fetal development and as little time duration as possible for pregnant women’ comfort [7,8]. But the fundamental reason is the imaging ability of MRI hardware.

As we know, low field MRI systems are still popular in China and many other countries. It is desirable to achieve MRI images with high quality, like images acquired from high field MRI. Image interpolation plays an important role for this scenario, since HR images can provide convincing information to observe fetal spine development, to detect abnormality and to classify malformations. The major advantage of image interpolation is that it may cost less and the existing low-end imaging systems can be still utilized. With sufficient messages, doctors are able to make correct prenatal diagnosis, design proper treatment planning or fetal surgery if necessary [5-8].

Image interpolation has been widely applied in different medical modalities [9-11], even problems exist. These problems are highly related to image edges, including the blurring of sharp edges, blocking artifacts in diagonal edges and inability to generate fine details [9-12]. Image interpolation can be classified into linear spatially invariant interpolation [9-12], transform domain interpolation [13,14], statistical learning based interpolation [15,16] and edge adaptive interpolation [17-28]. The HR images generated by these approaches are not as good as we expected. A fundamental deficiency of these approaches is that strong dependencies among pixels in an image are tacitly ignored. These dependencies pose important information about the anatomical structures, such as shape, texture, *et al*[29-31]. For the importance of edge preservation, edge adaptive interpolation approach becomes a center of focus.

A number of EDI methods have been presented [17-28]. EDI methods aim for generating HR images by taking edge information into consideration. The first EDI algorithm is based on two steps, rendering phase and correction phase [19]. It smoothes parallel to edges estimated by a subpixel estimation technique, but tends to be overly sensitive to produce noisy artifact in HR images. *Li*[20] proposed a new edge-directed interpolation (NEDI) which uses geometric duality to estimate covariance of HR area from that of local window pixels in LR image. By the fourth-order linear interpolation, it obtained a HR image with resolution of 2^*n*^ times to that of the LR image.

The basic assumption of NEDI is that there are significant correlation between LR and HR image which is inadequate and easy to introduce artifacts in high frequency region. Asuni and Giachetti, 2008 [27] discussed several problems in NEDI and analyzed from window shape, edge pixel handling, error propagation to global brightness invariance. Improved NEDI provides better results at the cost of huge computational complexity and parameter-dependent. Tam et al., 2010 [28] proposed a modified version of NEDI which adopted a modified training window structure to eliminate the predication accumulation problem and extended the covariance matching into multiple directions to suppress the covariance mismatch problem. The theory of NEDI is a least-squares estimation of neighborhood patterns, and [26] introduced non-local means as a weighting method to obtain robust improvement used in multi-valued diffusion weighted images. These methods overcome defects and restrict error propagation [26-28] of NEDI with the cost of higher computational consumption.

The second disadvantage of NEDI is its high computational complexity which restricts its capacity in real-time applications. Many EDI methods aim for decreasing time consumption. *Zhang*[22] reduced time cost and ringing artifacts via directional filtering and data fusion (EGII). Muresan, 2005 [24] detected the presence of edges, and classified the EDI procedure into diagonal and non-diagonal. It clarified its competitive speed to that of polynomial interpolation, but the edge direction classification is dependent on hard threshold. *Shi*[21] used Canny operator to detect edges from the pre-interpolated HR image by bilinear or bi-cubic, and applied Sobel horizontal and vertical derivative to determine the edge orientation and then modified these neighbors around edge points (CEM). *Andrea*[23] proposed ICBI method for real-time application with artifact-free. These methods [22-24] reduce time consumptions by introducing edge-directed idea into traditional method and simplifying estimation procedure.

The last disadvantage of NEDI may be its 2^*n*^ integer enlargement factor. In real applications to investigate a region of interest, image magnification procedure is step by step. From this aspect, many EDI approaches [20,22,23,26-28] are no better than traditional methods [9-11]. Meanwhile, the error will dynamically propagate forward during iteration which may result in the distortion. Strategies to restrict error propagation and enhance robustness should be taken into consideration.

## Method

This section focuses on the proposed true edge-directed interpolation (TEDI). We call it “true” because edge information is automatically extracted by a robust and accurate detector Canny. Meanwhile, the magnification factor varies step by step which distinguishes TEDI from many other EDI methods. TEDI mainly includes two parts. One is to evaluate edge information of edge map and orientations of edge points from Canny operator and the other is to soften or sharpen pixels on edge map with optimal blending weights.

### True edge-directed interpolation

Edge carries heavy structural information for human vision system which leads to detection, classification and decision. Edge detection in general is to preserve the structure properties with reduced amount of data. Canny detector [32] is one of the standard detection methods for its effectiveness, accuracy and robustness [21,32-34]. As compared to other edge detectors, it satisfies three criteria. First, it maximizes the probability to mark real points and reduces the number of non-edge points (good detection). Second, detected edge points are close to the center of edge (good localization). Finally, the detected edges are of one pixel width [34]. Orientations of edge points can be classified into four categories. Taking these superiorities into consideration, we adopt Canny detector to calculate edge map and orientations of edge points. The output edge map contains edge points with high probability and weak edge points which are 8-connected to the real edge. Orientations of edge points can be divided into 4 parts, 0° to 45° as right-horizontal (*RH*), 45° to 90° as left-vertical (*LV*), 90° to 135° as right-vertical (*RV*), and 135° to 180° as left-horizontal (*LH*). And we have formulations that:

(1) EM=LH⋃RV⋃LV⋃RH

(2) ∅=LH⋂RV=LH⋂LV=LH⋂RH=RV⋂LV=RV⋂RH=LV⋂RH

**Figure 1:**
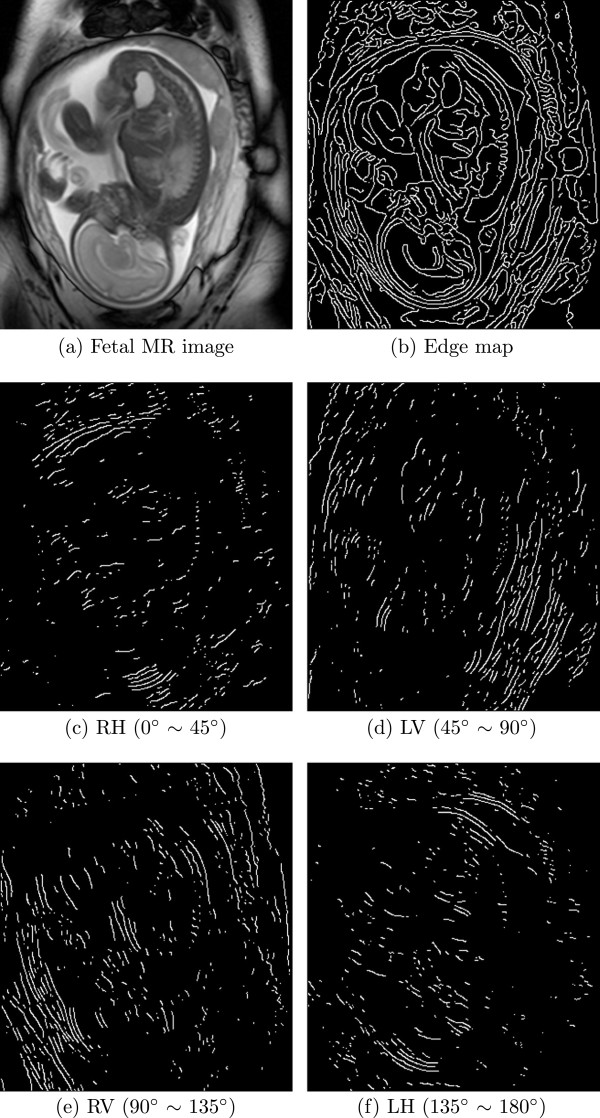
**Edge map and orientations of edge points. (a)** is the fetal spine MR image. Its resolution is 512 ×448. **(b)** is edge map extracted by Canny detector. *RH ***(c)**, *LV ***(d)**, *RV ***(e)** and *LH ***(f)** are shown with binary value.

Figure 1 shows an edge map and orientations of edge points. (a) is a fetal spine MR image. Image resolution is 512 ×448. (b) is the edge map from Canny detector with threshold 0.1. The other four subfigure show edge points in different categories.

**Figure 2:**
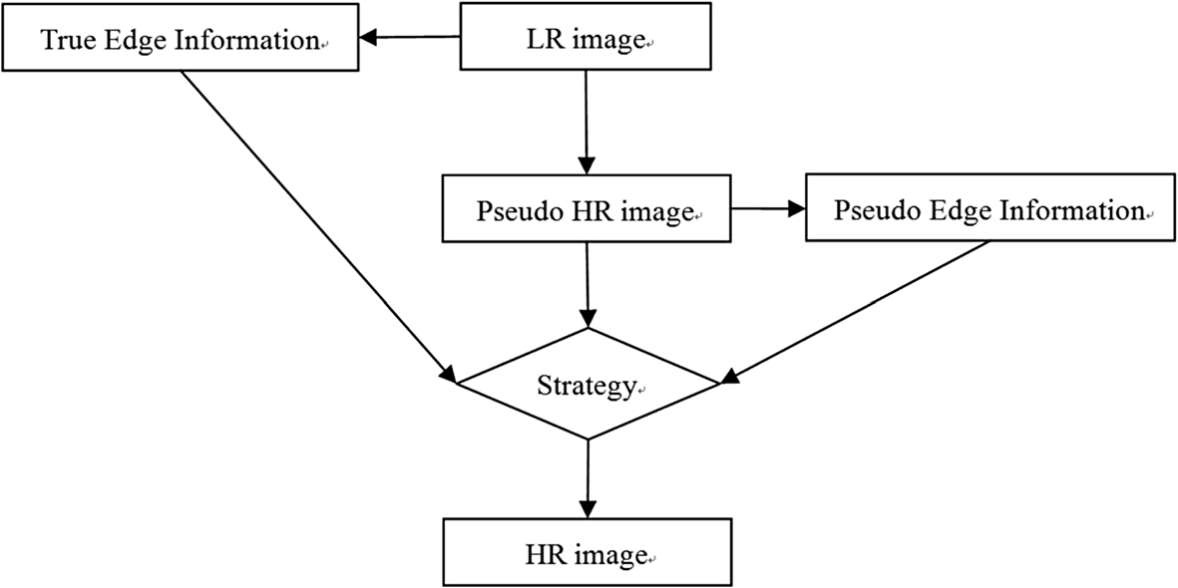
**Work flow of proposed TEDI algorithm.** Work flow of proposed TEDI method. Based on pseudo HR image, TEDI compares edge information with LR image to determine the strategy of sharpening or softening.

The proposed TEDI method calculates orientations of edge points for sharpening edges and reducing annoying blocking. We believe that edges existing in LR images should be reflected in corresponding HR images, and few errors induced in interpolation are more reliable in image analysis and medical diagnosis. Figure 2 presents the work flow of TEDI. The emphasis of TEDI is how to utilize edge messages to sharpen or soften edge points. Firstly, LR image can be interpolated into pseudo HR image for arbitrary size by any traditional interpolation methods [9,10]. For comparing with other four EDI methods, we set magnification factor as 2. Then we apply Canny operator to detect edge information for refining the pseudo HR image. True and pseudo edge information are both taken into strategy for different purposes. The strategy includes two parts. One is to sharpen edge points extracted from LR image, and the other is to soften edges from HR image.

### Determination of blending weights

**Figure 3:**
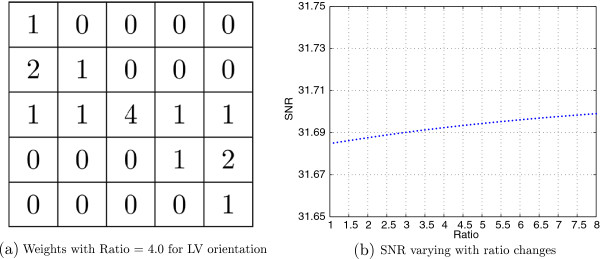
**Optimal blending weights determination.** Optimal blending weights determination. **(a)** is an instance for ratio-involving weighting matrix with ratio 4.0, and **(b)** describe SNR varying with ratio changes.

For softening edges from HR image, we simply average the 8 neighbor points to determine the points on the pseudo edge map. For sharpening the edge points on the true edge map, an optimizing procedure is proposed to find proper blending weights. There are 24 neighbors for a point *f*(*i*,*j*) in a 5 ×5 matrix. If it’s an edge point with known orientation *LV*, its blending weights will be symmetrically masked as shown in Figure 3a. Suppose the point *f*(*i*,*j*) is weighted with ratio, points of *f*(*i*−1, *j*−2) and *f*(*i*+1, *j*+2) are weighted with square root of ratio. Figure 3b is average SNR varying with respect to ratio change based on standard images from the website [35]. When maximizing SNR, we found that when ratio larger than 4.0, the average SNR of interpolated images is increasing slowly less than 0.01 dB. In this paper, the optimal blending weights we chose is a 2D designed filter of 5 ×5 with ratio equal to 4.0.

### Ethical approval

In this study we analyzed 12 fetal spine MR images from 3 pregnant participants, which was approved by the Institutional Review Board of Shenzhen Institutes of Advanced Technology. Written informed consent was obtained for all participants. These data could only be accessed to the physicians and researchers to ensure participant confidentiality.

## Experiment

In this section, we first describe materials and software used in the experiments, and then depict metrics for evaluating the image quality. The experiment focuses on five EDI methods applied in fetal spine MR images. They are NEDI [20], CEM [21], EGII [22], ICBI [23], and the proposed TEDI, respectively.

### Materials

All fetal spine MR images are imaging on a 1.5T scanner (SIEMENS, Sonata) with T2* GR scanning sequences (TR/TE: 3.63/1.82ms; FA: 71 degrees; FOV: 339 mm × 388 mm; the acquisition matrix: 512 ×448, region of interest is 360 ×320). The physical resolution is 0.662 mm × 0.866 mm. Fetal movement results in motion artifacts. These inevitable distortions in image acquisition may influence interpolated results from these EDI methods where error propagation can’t be restricted.

### Software

Codes of NEDI, EGII and ICBI are from [36-38] with no modification. Implementation of CEM follows the idea [21] and the threshold is 0.05. The pseudo HR image of CEM and TEDI are both interpolated by bi-linear which is implemented as a parameter in function *imresize* in MATLAB2008. All codes are running on a PC with Intel (R) Core (TM), i3-2120 CPU @ 3.30 GHZ, 3.29 GHZ, and 1.98 GB DDR RAM.

### Metrics

Performances are evaluated from SNR, PSNR, SSIM [29], FSIM [31], MI [39] and TC. Image quality assessment is so difficult [30] that we select 5 parameters to evaluate. SNR and PSNR are from error sensitivity based model, and widely used as objective image distortion metrics. SSIM and FSIM measure structure information maintenance from pixel level and feature level. MI is a basic concept of information theory to measure the statistical dependence and the amount of information. For clinical requirements, we introduce TC to measure real-time ability.

We also analyze visual quality of fine structures from interpolated images respecting to its practical procedures. Visual analysis is the only correct but burdensome way to distinguish image quality with sharp edges, natural textures and freedom from errors. They are three main criteria in the perceived quality of the interpolated images.

### Experiment design

**Figure 4:**
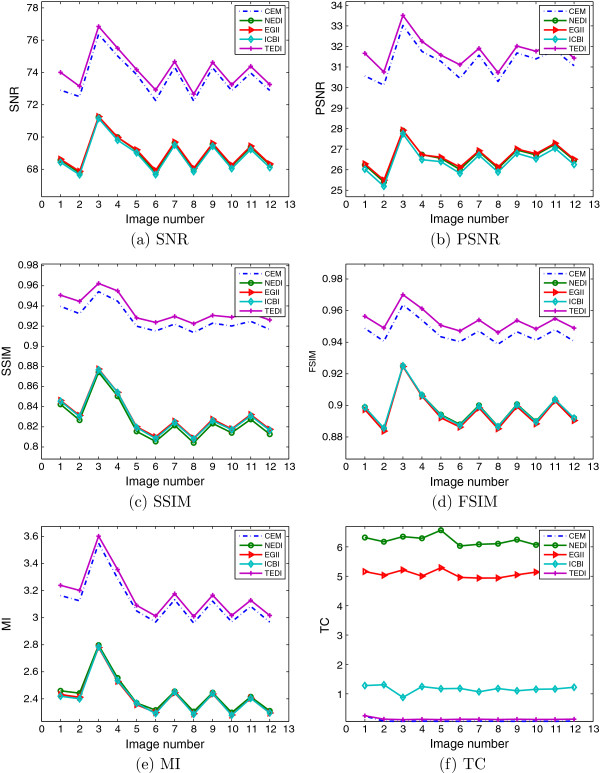
**Values of six metrics to fetal spine MR images. (a-f)** show SNR, PSNR, SSIM, FSIM, MI, and TC of these five EDI methods, respectively.

Performance is evaluated from quantitative metrics and visual analysis. Experiments are designed into two groups. The first group is from quantitative assessment. The source images are set as standard. For comparison, the standard images are scaled to 50% proportion with nearest neighbor pixel replication method. The results are evaluated by 6 parameters and demonstrated in Figure 4. The average scores are calculated to show methods’ robustness in Table 1. The second group is from visual analysis. We firstly down-scale the standard images by 0.5 with nearest neighbor pixel replication method and then enlarge them by a factor 4. In clinical applications, high visual quality with no error induced is appreciated for accurate image analysis and correct medical diagnosis.

**Table 1:** Unbiased metrics for five EDI methods

	**NEDI**	**CEM**	**EGII**	**ICBI**	**TEDI**
SNR	68.97 ± 1.05	73.63 ± 1.25	69.03 ± 1.03	68.83 ± 1.05	**74.12** ± 1.21
PSNR	26.60 ± 0.64	31.25 ± 0.82	26.65 ± 0.63	26.41 ± 0.65	**31.74** ± 0.75
SSIM	0.82 ± 0.02	0.93 ± 0.01	0.83 ± 0.02	0.83 ± 0.02	**0.94** ± 0.01
FSIM	0.90 ± 0.01	0.95 ± 0.01	0.90 ± 0.01	0.90 ± 0.01	0.95 ±0.01
MI	2.43 ± 0.14	3.11 ± 0.17	2.41 ± 0.14	2.41 ±0.14	**3.17** ± 0.17
TC	6.26 ± 0.06	**0.08** ± 0.05	5.09 ± 0.08	1.16 ± 0.12	0.14 ± 0.02

## Results

### Image quality assessment

This section illustrates the SNR/PSNR, SSIM/FSIM, and MI/TC of five EDI methods. SNR and PSNR of TEDI are higher than CEM and outperform NEDI, EGII and ICBI with more than 4.5 dB. TC of TEDI and CEM are much shorter than other three EDI methods. SSIM, FSIM and MI of TEDI are slightly higher than others. From Figure 4, it can conclude that TEDI is better than the other EDI methods. Due to its real-time implementation, the proposed method satisfies clinical requirements.

Unbiased evaluations are summarized as average value and standard deviation in Table 1. From Table 1, it can conclude that: TEDI and CEM win by 5.0 db and 4.6 db respectively to other EDI methods; TEDI outperforms CEM slightly and other methods with 0.1 from SSIM, and 0.05 from FSIM; MI amount from TEDI is promoted from 0.06 to 0.76, and CEM is with least time consumptions.

### Visual quality analysis

**Figure 5:**
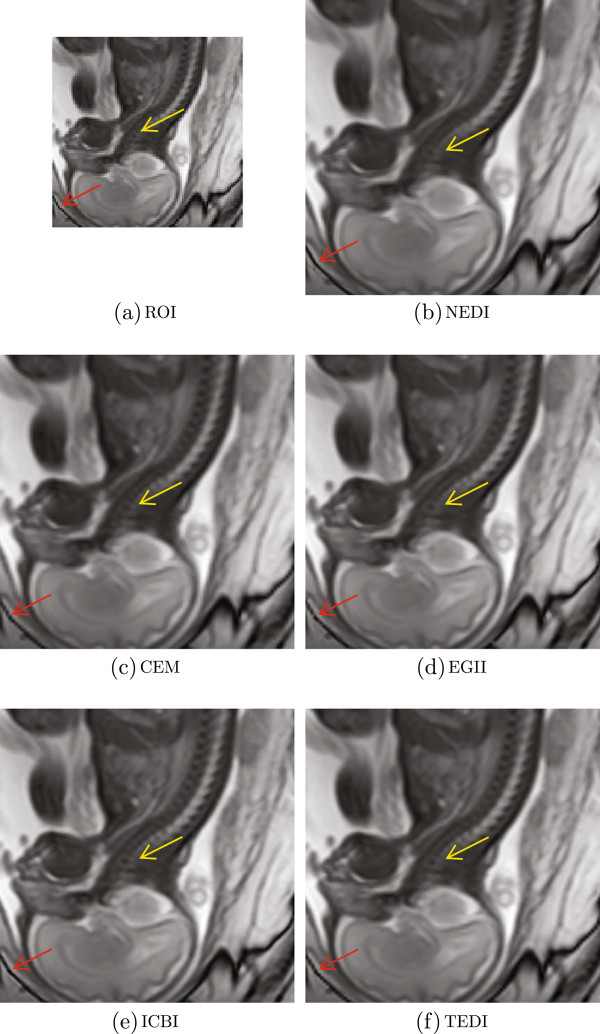
**Visual analysis of structures in ROI. (a)** is original fetal spine MR image, **(b-f)** stand for the interpolated results from NEDI, CEM, EGII, ICBI, and TEDI, respectively. Because of low quality in **(a)**, unnatural textures, loss of edge sharpness and errors are seen. Interpolated result from TEDI shows better consistency with the original image.

Figure 4 shows that the 2 ^*nd*^ image is with lowest SNR, PSNR and FSIM, and visual analysis is based on this image. In this time, the image is zoom-in by 50% proportion with nearest neighbor pixel replication method. Different from the quantitative assessment, we interpolated it with enlargement factor of 4, and the resolution of interpolated results will be 720 ×640. Meanwhile, two ROI are delineated to demonstrate visual details in Figure 5 directed by red and yellow arrows.

Figure 5 demonstrates interpolated results from these five EDI methods. Visual quality is analyzed from three criteria, sharpness of edges, naturalness of textures and no errors. (a) In the original image, there are obvious staircase edges directed by the red arrow. Because of low resolution, the intensity transmission isn’t smooth enough from bright side in cervical-medullary junction to dark side in cervical spine directed by yellow arrow. (b) Since no restriction in error propagation, whirlpool-like textures, ringing artifacts and outliers are seen in results from NEDI. Yellow arrow indicates improper intensity transmission from bright to dark, and red arrow indicates outliers. (c) CEM takes every pixel on edges to be vertical or horizontal direction. The ROI directed by yellow is deteriorated by artifacts and blurring. (d) EGII applies statistical predication to achieve more robust estimation. The yellow arrow shows that the intensity transmission of fetal spine is disturbed by an expanded black circle where artifacts of annoying ambiguous blocking occur. In addition, discontinuities appear at the head of the red arrow. (e) ICBI restricts artifacts and errors, and arrow-directed regions show sharper edges and natural transmission from bright to dark. (f) TEDI classifies edge orientation into four categories and sharpens edges with optimal blending weights. The interpolated image well maintains consistent structures. The yellow arrow indicates smooth intensity transmission from bright to dark. Edge structures directed by red arrow can be observed with no outliers nor errors.

## Discussion

Ultrasound remains the primary modality for evaluating the developing fetus, and MRI of the fetal spine is complementary, even superior when ultrasound fails to provide further details for correct diagnosis. MR images of fetal spine suffer from its low resolution and image interpolation becomes necessary for better readability. In clinical situations, motion artifacts [10,40-42] from respiratory motion, partial volume effects and non-uniform magnetic field strength are prevalent in MR images. Interpolating unknown pixels in HR image from those known ones in LR image is an inverse ill-posed problem. Wrong predications should be seriously considered and tackled accordingly.

Edge carries heavy messages for accurate diagnosis while many interpolation methods fail to preserve. For the importance of edge preservation, EDI methods become a center of focus. In this paper, we proposed a TEDI method from twofold considerations. One is to determine the edge information which includes edge map and orientations of edge points. The other is a strategy to soften or sharpen the pixels on edge maps with respect to its orientation.

From performance evaluations of five EDI methods, we find that all the EDI methods can generate HR images with enriched structural details. From objective assessment, the proposed TEDI outperforms the other four methods with a seconds-level time cost. From visual quality analysis of image structures in ROI, only ICBI outperforms the TEDI with crisper edges. Both NEDI and EGII introduce errors and outliers, and artifacts is magnified by NEDI. In these five EDI methods, NEDI, EGII and ICBI share similar interpolation procedure. The difference is how to fill the value of missing pixels in HR image. NEDI uses geometric duality and takes fourth-order linear interpolation. EGII takes statistical prediction with linear minimum mean square-error estimate technique. ICBI selects local approximations and a greedy minimization. Indeed, NEDI is superb with higher number of directions over TEDI and it can adapt to arbitrary directions, because covariance can be computed for any axis orientation. But for real applications, maybe simpler is better. For magnifying medical images from low-end systems, four edge categories are enough. On the one hand, in original images, neighboring pixels around the pre-interpolated pixel position may be inadequate and inaccurate. On the other hand, computing covariance in NEDI imposes large time consumptions. In addition, experimental results show that NEDI’s adoption to arbitrary directions damages image visual quality with distorted textures and magnified artifacts.

One disadvantage to be mentioned is the selection of metrics for objective evaluation. These metrics except TC are based on reference images. Since motion artifacts and noise are prevalent in medical images, it is difficult to acquire high-quality fetal MR images on a low field MRI system as reference. In addition, these inevitable distortions may result in inaccurate prediction and errors in EDI algorithms. This kind of mistakes will be magnified in consecutive iterations.

## Conclusions

hjbImage interpolation is intrinsically a severely under-determined inverse problem, and aims to resolve unknown HR images from known LR images [9-12]. Artifacts from respiratory motion [10,40], elicited or spontaneous movement of fetus, partial volume effects [41] and non-uniform magnetic field strength [42] pose significant changelings for medical diagnosis which degrade image quality and mislead interpolation. Fully interpolation from LR images for high visual quality is still changeling for these existing methods.

In this paper, a new interpolation method is proposed. Comparing with other four state-of-the-art EDI methods, the proposed TEDI outperforms the others from objective evaluation. From visual analysis, NEDI, CEM and EGII are not suitable for clinical applications since their sensitivity to noise and artifacts. ICBI and TEDI are good choices, and TEDI is better in preserving structures consistent with original images.

In medical images, artifacts and noise are prevalent. A preprocessing of artifact removal and noise suppression should be taken into account before image interpolation. Image interpolation may be useful to improve the readability of fetal MR images, but it inevitably introduces uncertainties in image content. Restricting these uncertainties during interpolation procedure is also necessary.

## Abbreviations

MRI: Magnetic resonance imaging; EDI: Edge-directed interpolation; HR: High-resolution; LR: Low-resolution.

## Competing interests

The authors declare that they have no competing interests.

## Authors’ contributions

SY: proposed the idea, performed experiments, analyzed the data, made discussions and composed the manuscript together with RZ, SW. JH: provided fetal spine MR images and made the discussions. YX: directed the experiments and made discussions. All authors read and approved the final manuscript.
